# Supplementary material for: Analysis of Volatile Compounds in Pears by HS-SPME-GC×GC-TOFMS
Source: Molecules. 2019 May 9;24(9):1795. doi: 10.3390/molecules24091795 (PMC6539139; doi:10.3390/molecules24091795)
Supplement: Supplementary file 1 [file molecules-24-01795-s001.pdf]

Supplementary:

Analysis of Volatile Compounds in Pears by HS-SPME-GC×GC-TOFMS

Chenchen Wang [1,2], Wenjun Zhang [1,2], Huidong Li [1,2], Jiangsheng Mao [1,2], Changying Guo [1,2], Ruiyan Ding [1,2], Ying Wang [3], Liping Fang [1,2], Zilei Chen [1,2,4,*] and Guosheng Yang [5,*]

Table S1. The peak numbers of different classes obtained using 4 SPME fibres.

	50/30 µm DVB/CAR/PDMS	85 µm PA	65 µm PDMS/PVB	100 µm PDMS
Esters	67	49	62	46
Alkenes	19	3	27	12
Alkanes	19	4	17	14
Arenes	20	2	15	0
Aldehydes	9	6	10	2
Ketones	5	2	4	4
Alcohols	9	7	7	4
Others	15	8	4	2
Total	163	81	146	84

Table S2. Concentrations of volatile compounds in pear cultivars.

No.	Volatile compounds	[1]R.T. (s)	[2]RI	Longyuanyangli (ng/g)	Packham (ng/g)	D Jules (ng/g)	Clapp (ng/g)	Stark (ng/g)
Esters (101)								
1	Methyl acetate	102, 0.630	500.4	[3]16.22±0.56	19.70±3.48	13.97±5.44	13.85±10.19	14.30±0.32
2	Ethyl Acetate	132, 0.810	605.1	66.94±11.81	10.81±5.74	58.90±22.34	48.3±0.79	39.44±41.09
3	Methyl propionate	138, 0.760	614.6	1.55±0.14	[4]-	-	-	-
4	Ethyl propanoate	198, 0.900	705.3	16.64±5.85	-	-	-	-
5	n-Propyl acetate	201, 0.920	707.9	111.27±3.60	2.87±1.91	95.40±19.72	24.73±1.23	59.64±0.42
6	Methyl butanoate	210, 0.930	715.6	11.59±1.77	0.35±0.13	-	-	0.08±0.02
7	Ethyl-2-methylproanoate	255, 0.930	754.0	0.94±0.70	-	-	-	-
8	Methyl (E)-2-butenoate	261, 1.120	759.4	0.69±0.08	-	-	-	-
9	Methyl 2-butenoate	264, 1.110	761.9	-	-	-	-	0.03±0.01
10	2-Methylpropyl acetate	273, 0.970	769.4	8.43±0.61	0.46±0.29	5.48±0.75	3.66±0.17	3.64±0.30
11	Methyl 2-methylbutanoate	279, 0.980	774.6	2.62±0.27	0.49±0.03	0.26±0.04	0.50±0.09	0.35±0.08
12	Ethyl butyrate	312, 1.080	802.0	36.37±6.88	-	-	-	-
13	Butyl acetate	330, 1.130	812.7	75.90±53.77	54.69±15.25	17.46±21.05	39.93±9.65	8.90±4.98

14	Methyl valerate	348, 1.100	823.4	0.73±0.08	-	-	-	-
15	Ethyl (Z)-2-butenoate	381, 1.250	843.1	3.66±0.22	-	-	-	-
16	Ethyl 2-methylbutyrate	390, 1.080	848.3	18.01±4.15	0.05±0.01	0.12±0.05	0.13±0.04	-
17	2-Methyl-1-butyl acetate	441, 1.130	878.8	60.23±2.89	-	8.21±1.02	5.19±0.39	4.32±0.33
18	3-Buten-1-ol, 3-methyl-, acetate	450, 1.200	884.2	0.07±0.01	-	-	-	0.19±0.12
19	Propyl butanoate	474, 1.140	898.4	1.50±0.02	-	-	-	-
20	Ethyl pentanoate	477, 1.160	900.2	0.83±0.11	-	-	-	-
21	Butyl propanoate	492, 1.160	908.4	0.08±0.01	-	0.37±0.08	0.36±0.08	0.26±0.02
22	Pentyl acetate	504, 1.190	915.0	68.9±4.11	7.08±5.82	24.91±7.19	21.47±3.87	21.54±2.02
23	Butyrolactone	513, 2.620	920.7	0.22±0.21	-	1.86±0.06	-	-
24	Methyl hexanoate	522, 1.230	924.8	80.75±13.36	1.02±0.27	0.53±0.12	0.61±0.07	1.25±0.17
25	Methyl (E)-3-hexenoate	537, 1.290	933.0	0.05±0.01	-	-	-	-
26	Methyl (Z)-3-hexenoate	543, 1.320	936.3	0.08±0.01	-	-	-	-
27	Ethyl tiglate	549, 1.310	939.6	0.19±0.02	-	-	-	-
28	Propyl 2-methylbutanoate	561, 1.130	946.1	0.55±0.12	-	-	-	-
29	Butyl 2-methylpropanoate	576, 1.120	954.3	-	-	-	0.18±0.04	0.06±0.01
30	2-Methylpropyl butyrate	579, 1.140	955.9	0.12±0.00	-	-	-	-
31	Methyl 2-hexenoate	597, 1.360	965.9	0.28±0.10	-	-	-	-
32	2-Methylpentyl acetate	624, 1.180	980.5	-	-	-	-	0.67±0.09
33	Vinyl caproate	633, 1.270	985.5	0.12±0.04	-	-	-	-
34	Butyl butyrate	654, 1.190	996.9	0.46±0.05	0.06±0.04	0.68±0.09	3.35±0.77	1.84±0.27
35	Ethyl hexanoate	660, 1.270	1000.2	37.76±1.82	-	0.06±0.01	0.08±0.02	0.12±0.02
36	Isobutyl 2-methylbutanoate	669, 1.100	1005.0	-	-	0.02±0.01	0.18±0.02	0.01±0.00
37	(Z)-3-Hexenol acetate	672, 1.280	1006.7	5.41±0.38	1.03±0.13	8.51±0.56	10.98±0.30	6.02±3.89
38	Ethyl (E)-3-hexenoate	681, 1.310	1011.5	0.74±0.05	-	-	-	-
39	Hexyl acetate	690, 1.370	1016.3	9.99±8.97	51.58±7.86	49.52±38.9	44.32±11.96	5.26±3.59
40	(Z)-2-Hexenyl acetate	693, 1.360	1018.0	3.65±2.01	11.46±6.36	2.66±2.61	11.99±0.98	16.84±2.18
41	Methyl 2,4-hexadienoate	693, 1.590	1018.1	0.28±0.08	-	-	-	-
42	(Z)-4-Hexen-1-ol acetate	702, 1.320	1024.4	-	-	0.74±0.07	0.85±0.07	0.56±0.03
43	Methyl heptoate	708, 1.240	1026.0	1.31±0.18	0.05±0.01	-	0.15±0.02	0.24±0.02
44	Butyl 2-methylbutanoate	741, 1.160	1043.7	0.25±0.07	-	0.41±0.11	3.43±1.45	0.84±0.22
45	Ethyl 2-hexenoate	744, 1.380	1045.4	0.51±0.07	-	-	-	-
46	2-Methylbutyl butanoate	771, 1.180	1059.8	0.31±0.04	-	-	0.06±0.01	-
47	1-Hexanol, 4-methyl-, acetate	816, 1.220	1084.0	0.18±0.02	-	0.07±0.05	-	0.19±0.03
48	(E,E)-Ethyl 2,4-hexadienoate	834, 1.560	1093.9	0.32±0.05	-	-	-	-
49	Butyl pentanoate	834, 1.240	1093.7	-	-	-	0.10±0.01	-
50	Propyl hexanoate	837, 1.240	1095.3	0.96±0.21	-	0.03±0.00	-	0.10±0.00
51	Methyl benzoate	840, 1.850	1097.3	0.16±0.02	-	-	0.05±0.01	0.14±0.07
52	Ethyl heptanoate	843, 1.240	1098.6	1.17±0.12	-	-	-	-
53	Ethyl	849, 1.730	1102.2	0.12±0.02	-	-	-	-

	3-(methylsulfanyl)propanoate							
54	2-Methylbutyl 2-methylbutanoate	855, 1.160	1105.2	-	-	-	0.20±0.04	-
55	3-Hepten-1-ol, acetate	855, 1.330	1105.2	-	-	-	-	0.22±0.04
56	Hexyl propanoate	858, 1.230	1107.0	-	-	-	1.53±0.28	0.83±0.30
57	Heptyl acetate	870, 1.260	1113.7	20.98±3.38	0.07±0.04	39.82±6.28	9.68±0.17	21.38±6.14
58	methyl 4-octenoate	876, 1.350	1117.2	0.47±0.12	-	-	0.05±0.00	0.09±0.01
59	Methyl octanoate	891, 1.280	1125.6	5.16±1.11	0.07±0.00	0.23±0.04	1.12±0.09	3.45±0.72
60	3-(Methylthio)propyl acetate	891, 1.810	1125.9	0.27±0.03	-	0.48±0.02	-	-
61	Methyl (Z)-3-octenoate	900, 1.380	1130.8	0.03±0.01	-	-	-	-
62	Hexyl 2-methylpropionate	933, 1.180	1149.3	-	-	-	0.15±0.04	-
63	2-Methylpropyl hexanoate	936, 1.200	1151.0	0.13±0.07	-	-	0.07±0.01	-
64	3-Methylheptyl acetate	936, 1.200	1152.7	-	-	0.37±0.05	-	1.05±0.31
65	Methyl (E)-2-octenoate	969, 1.420	1169.8	0.20±0.11	-	-	-	0.03±0.01
66	Ethyl benzoate	975, 1.800	1173.4	0.07±0.00	-	-	-	-
67	Methyl benzeneacetate	987, 2.000	1180.3	0.11±0.02	-	-	-	-
68	Ethyl 3-(methylthio)-(E)-2-propenoate	990, 1.970	1181.9	0.08±0.00	-	-	-	-
69	(Z)-3-Hexenyl butanoate	999, 1.320	1186.6	-	-	-	0.09±0.00	-
70	(Z)-Ethyl 4-octenoate	1005, 1.350	1190.1	0.97±0.26	-	-	-	-
71	Hexyl butanoate	1008, 1.260	1191.7	1.43±0.41	0.07±0.05	5.96±1.20	9.05±1.54	3.97±1.31
72	Butanoic acid, 2-hexenyl ester, (Z)-	1014, 1.350	1195.1	-	-	-	0.49±0.00	0.32±0.13
73	Ethyl octanoate	1020, 1.260	1198.5	8.26±2.05	-	0.09±0.01	0.24±0.05	0.43±0.15
74	(Z)-3-Octen-1-ol acetate	1020, 1.330	1198.5	-	-	0.79±0.13	-	0.11±0.01
75	Cyclohexylethyl acetate	1038, 1.370	1209.2	0.87±0.17	-	13.44±5.08	1.32±0.09	3.72±0.74
76	Octyl acetate	1044, 1.270	1212.7	1.04±0.33	-	23.94±10.88	5.24±1.18	14.46±5.34
77	Methyl nonanoate	1065, 1.280	1225.2	-	-	-	0.05±0.02	-
78	Hexyl 2-methylbutanoate	1086, 1.210	1237.6	0.42±0.15	0.03±0.00	0.21±0.03	0.55±0.12	0.21±0.08
79	Hexyl 2-methyl-2-propenoate	1095, 1.410	1243.1	-	-	-	0.02±0.00	0.04±0.01
80	Ethyl (E)-2-octenoate	1101, 1.410	1246.7	1.42±0.39	-	-	-	-
81	trans, trans-Octa-2,4-dienyl acetate	1101, 1.500	1246.7	-	-	0.25±0.05	0.18±0.01	0.27±0.12
82	Ethyl benzeneacetate	1101, 1.910	1247.0	0.32±0.03	-	-	-	-
83	2-Methylbutyl hexanoate	1113, 1.240	1253.7	0.16±0.04	-	0.03±0.00	0.11±0.01	0.03±0.01
84	2-Phenethyl acetate	1122, 1.900	1259.5	0.39±0.08	-	0.18±0.01	-	0.02±0.01
85	Hexyl n-valerate	1173, 1.260	1289.5	-	-	0.05±0.01	0.27±0.23	0.08±0.02
86	Propyl octanoate	1179, 1.260	1293.0	-	-	-	-	0.06±0.03
87	Methyl (Z)-4-decenoate	1206, 1.370	1309.9	0.04±0.01	0.04±0.03	-	0.01±0.00	0.02±0.01

88	Methyl decanoate	1230, 1.290	1325.2	0.12±0.03	0.10±0.08	0.23±0.07	0.09±0.01	0.19±0.08
89	6-Octen-1-ol, 3,7-dimethyl-, acetate	1275, 1.340	1354.1	-	-	0.14±0.01	-	-
90	Butyl benzoate	1311, 1.750	1377.4	-	-	-	0.01±0.00	-
91	Hexyl hexoate	1326, 1.270	1386.7	0.25±0.08	-	1.63±0.32	8.13±3.39	2.55±1.55
92	Ethyl decanoate	1341, 1.270	1396.3	0.11±0.03	-	-	-	-
93	Methyl (E,Z)-2,4-decadienoate	1344, 1.510	1398.4	0.09±0.02	0.53±0.32	-	-	-
94	Decyl acetate	1365, 1.270	1412.2	-	-	0.37±0.12	-	-
95	Ethyl (E,Z)-2,4-decadienoate	1452, 1.490	1470.3	0.12±0.04	0.05±0.05	-	-	-
96	Methyl dodecanoate	1533, 1.290	1525.7	-	-	0.22±0.08	0.15±0.03	0.34±0.16
97	Hexyl octylate	1614, 1.280	1583.2	-	-	-	0.11±0.05	0.05±0.02
98	Methyl (6E)-6-nonenoate	1770, 1.380	1698.0	-	-	0.09±0.03	-	-
99	Methyl tetradecanoate	1806, 1.300	1726.4	0.03±0.01	-	0.09±0.03	-	0.13±0.05
100	Methyl hexadecanoate	2052, 1.110	1933.5	0.04±0.01	-	0.12±0.05	0.16±0.06	0.12±0.05
101	Methyl (Z)-9-octadecenoate	2181, 0.840	2063.8	0.03±0.02	-	0.04±0.03	0.02±0.01	0.03±0.03
	Subtotal			692.72	162.66	378.94	273.54	241.03
Alkenes (30)								
102	1,4-Pentadiene	108, 0.580	522.4	-	0.23±0.05	-	0.17±0.03	0.14±0.02
103	(Z),(Z)-2,4-Hexadiene	144, 0.660	623.9	-	0.28±0.11	-	-	-
104	1-Methyl-1,4-cyclohexadiene	255, 0.880	754.0	-	-	0.28±0.12	-	0.08±0.01
105	Styrene	459, 1.370	891.4	0.46±0.03	-	0.29±0.06	0.06±0.02	0.08±0.01
106	β-myrcene	645, 1.060	991.9	0.02±0.00	-	-	-	-
107	L-Limonene	714, 1.140	1030.8	0.31±0.03	-	-	-	-
108	1-Propenylbenzene	729, 1.550	1037.5	0.13±0.01	-	-	-	-
109	(Z)-β-ocimene	753, 1.130	1050.1	-	-	0.03±0.00	-	-
110	1-Ethenyl-3-ethylbenzene	822, 1.460	1087.4	0.06±0.02	-	0.24±0.08	0.05±0.01	-
111	1-Ethenyl-4-ethylbenzene	834, 1.480	1093.8	0.17±0.09	-	0.26±0.04	0.06±0.00	0.07±0.01
112	1,3-Diethenylbenzene	873, 1.610	1115.6	-	-	0.07±0.02	-	-
113	1,4-Diethenyl benzene	894, 1.660	1127.5	0.02±0.00	-	0.06±0.01	-	-
114	(Z)-3-Tetradecene	1176, 1.010	1291.1	-	-	0.04±0.01	-	-
115	α-Cubenene	1275, 1.180	1354.0	-	0.01±0.00	-	0.09±0.03	-
116	Copaene	1320, 1.240	1382.8	-	0.39±0.05	0.26±0.02	-	0.05±0.02
117	1-Tetradecene	1335, 1.040	1396.2	-	-	0.06±0.03	-	-
118	Aromandendrene	1368, 1.370	1414.2	0.07±0.02	-	-	0.03±0.00	-
119	(5Z)-2,6,10-Trimethyl-1,5,9-undecatrien	1431, 1.170	1456.1	-	-	0.14±0.03	-	-
120	(E)-β-Farnesene	1437, 1.240	1460.1	0.34±0.03	-	0.39±0.06	0.11±0.01	0.10±0.05
121	α-Humulene	1440, 1.410	1462.3	-	0.02±0.00	-	0.04±0.01	-
122	2,3,5,8-Tetramethyl-1,5,9-de	1446, 1.190	1466.1	0.16±0.03	-	0.27±0.10	-	-

	catriene							
123	(Z,E)-α-Farnesene	1494, 1.290	1498.2	3.64±1.41	-	5.50±0.96	0.73±0.04	1.02±0.28
124	α-Muurolene	1506, 1.400	1506.7	-	0.07±0.00	0.02±0.00	-	-
125	α-Farnesene	1518, 1.350	1512.9	15.78±12.87	0.04±0.03	15.07±3.89	15.10±2.94	17.47±2.99
126	(Z,Z)-α-Farnesene	1539, 1.290	1530.0	1.18±0.70	-	0.23±0.03	0.08±0.07	0.09±0.06
127	(+)-δ-Cadinene	1539, 1.420	1530.1	-	0.10±0.00	0.16±0.02	0.15±0.05	0.04±0.02
128	cis-Calamenene	1539, 1.570	1530.1	-	0.02±0.00	-	-	-
129	α-Calacorene	1569, 1.670	1551.5	-	0.01±0.00	0.03±0.00	-	-
130	(E)-γ-Bisabolene	1578, 1.340	1557.7	0.55±0.21	-	0.36±0.05	0.08±0.01	0.07±0.00
131	(Z)-γ-Bisabolene	1602, 1.360	1574.7	0.64±0.25	-	0.37±0.04	0.10±0.01	0.09±0.02
	Subtotal			23.53	1.17	24.13	16.85	19.3
Alkanes (12)								
132	Undecane	846, 0.890	1100.0	0.06±0.02	0.05±0.01	0.78±0.18	0.04±0.01	0.06±0.00
133	Dodecane	1023, 0.930	1200.0	0.17±0.04	-	0.19±0.04	0.05±0.00	0.06±0.01
134	2-Methyldodecane	1131, 0.930	1264.3	0.07±0.02	-	0.04±0.01	-	-
135	Tridecane	1191, 0.960	1300.0	0.91±0.26	0.05±0.01	0.55±0.17	0.15±0.04	0.16±0.02
136	2-Methyltridecane	1290, 0.960	1363.4	0.24±0.07	-	0.12±0.02	-	-
137	3-Methyltridecane	1302, 0.970	1371.1	0.24±0.06	-	0.12±0.03	0.03±0.01	0.03±0.00
138	Tetradecane	1347, 0.980	1400.0	1.87±0.67	0.17±0.04	1.54±0.22	0.58±0.17	0.43±0.13
139	3-Methyltetradecane	1455, 0.990	1472.0	0.16±0.04	-	-	-	-
140	Pentadecane	1497, 1.000	1500.0	0.88±0.32	0.13±0.07	1.91±0.55	0.33±0.03	0.36±0.08
141	2-Methylpentadecane	1587, 0.990	1563.8	0.16±0.06	-	-	0.05±0.00	-
142	Hexadecane	1638, 1.010	1600.0	0.65±0.21	0.06±0.03	0.81±0.20	0.22±0.02	0.19±0.06
143	Heptadecane	1773, 1.020	1700.0	0.12±0.05	-	0.15±0.04	0.05±0.01	0.11±0.02
	Subtotal			5.53	0.46	6.21	1.5	1.4
Arenes (19)								
144	Benzene	165, 0.800	657.4	-	-	0.28±0.12	-	-
145	Toluene	285, 0.990	761.8	0.85±0.15	0.35±0.35	0.76±0.11	0.40±0.03	0.36±0.04
146	Ethylbenzene	408, 1.190	859.2	0.87±0.04	-	-	0.05±0.02	0.06±0.01
147	p-Xylene	420, 1.200	866.3	10.74±3.68	0.30±0.20	8.22±0.28	2.99±0.33	2.77±0.61
148	1,3-Dimethylbenzene	462, 1.270	891.3	4.11±0.36	-	-	0.06±0.02	0.04±0.03
149	o-Xylene	468, 1.100	894.8	0.27±0.20	-	-	0.06±0.02	-
150	1-Ethyl-4-methylbenzene	591, 1.270	962.5	0.54±0.23	-	0.35±0.03	0.17±0.05	0.10±0.02
151	3-Ethyl-1-methylbenzene	603, 1.250	969.1	0.23±0.05	-	0.28±0.02	-	-
152	1,2,4-Trimethylbenzene	648, 1.330	993.7	0.30±0.01	-	0.10±0.02	-	-
153	1,2,3-Trimethylbenzene	705, 1.410	1024.5	0.60±0.06	-	-	-	-
154	p-Cymene	708, 1.280	1026.0	0.15±0.02	-	-	-	-
155	1-Methyl-3-propylbenzene	756, 1.300	1051.8	0.11±0.00	-	-	-	-
156	1-Methyl-2-propylbenzene	771, 1.320	1059.9	0.08±0.01	-	-	-	-
157	1,2-Dimethyl-4-ethylbenzene	819, 1.370	1085.7	0.08±0.03	-	-	-	-

158	1,2,3,4-Tetramethylbenzene	882, 1.440	1120.6	0.06±0.02	-	-	-	-
159	Naphthalene	996, 2.030	1185.4	0.58±0.09	-	0.34±0.06	0.22±0.03	0.21±0.03
160	2-Methylnaphthalene	1185, 2.000	1297.0	0.12±0.02	-	0.10±0.02	0.04±0.00	0.04±0.01
161	1-Methylnaphthalene	1212, 2.080	1314.2	0.05±0.01	-	0.04±0.01	0.01±0.00	0.02±0.01
162	1-(1,5-Dimethylhexyl)-4-methylbenzene	1422, 1.320	1452.0	0.09±0.02	-	-	0.03±0.00	-
	Subtotal			19.83	0.65	10.47	4.03	3.6
Aldehydes (28)								
163	Acetaldehyde	78, 2.100	417.2	23.71±8.76	5.01±2.71	4.81±1.91	13.10±3.36	10.19±5.33
164	Butanal	120, 0.720	567.3	0.52±0.05	0.23±0.05	1.58±1.23	0.52±0.13	0.38±0.06
165	(E)-2-Butenal	159, 0.910	648.1	-	-	0.60±0.71	-	-
166	Pentanal	192, 0.910	700.2	-	0.14±0.02	0.09±0.03	0.21±0.02	-
167	(E)-2-Methyl-2-butenal	237, 1.120	738.8	0.28±0.10	-	-	-	-
168	3-Methyl-2-butenal	291, 1.340	787.7	0.04±0.01	-	0.07±0.01	0.03±0.01	-
169	Hexanal	309, 1.160	800.2	12.46±8.42	5.63±0.77	2.07±0.16	2.68±0.48	2.78±0.05
170	3-Hexenal	312, 1.150	802.0	-	-	-	0.10±0.05	0.17±0.02
171	(Z)-2-hexenal	384, 1.310	846.7	-	-	-	0.06±0.01	1.98±2.57
172	(E)-2-Hexenal	393, 1.370	850.4	6.27±2.56	5.27±0.90	1.60±0.07	4.84±2.07	2.73±1.88
173	Heptanal	480, 1.240	901.9	-	0.03±0.01	0.07±0.01	0.08±0.02	0.04±0.01
174	(E,E)-2,4-Hexadienal	495, 1.630	910.3	0.10±0.02	0.13±0.01	-	0.06±0.00	0.10±0.02
175	(E)-2-Heptenal	579, 1.420	957.7	0.03±0.01	-	0.23±0.01	0.21±0.02	0.13±0.05
176	Benzaldehyde	588, 1.910	961.2	0.29±0.04	0.04±0.01	0.25±0.10	0.08±0.00	0.08±0.01
177	Octanal	666, 1.290	1003.5	0.27±0.13	0.04±0.01	0.34±0.15	0.24±0.12	-
178	(Z)-2-Octenal	717, 1.180	1030.8	0.38±0.25	-	0.44±0.06	0.64±0.05	0.37±0.03
179	Benzeneacetaldehyde	744, 2.040	1045.8	0.10±0.01	-	-	-	-
180	(E)-2-Octenal	768, 1.450	1058.4	0.13±0.03	0.03±0.01	0.61±0.05	0.43±0.05	0.31±0.12
181	Nonanal	855, 1.310	1105.3	-	0.07±0.00	-	1.14±0.55	-
182	(E,Z)-2,6-Nonadienal	942, 1.560	1154.6	-	-	-	0.05±0.01	-
183	(E)-2-Nonenal	954, 1.450	1161.3	-	0.06±0.02	0.88±0.18	1.10±0.80	0.53±0.28
184	cis-4-Decenal	1014, 1.410	1195.2	-	-	0.08±0.03	0.04±0.00	-
185	Decanal	1032, 1.330	1205.6	0.05±0.01	0.02±0.00	0.09±0.02	0.04±0.00	0.04±0.01
186	(E,E)-2,4-Nonadienal	1047, 1.630	1214.7	-	-	0.10±0.01	0.09±0.01	0.08±0.04
187	4-Methoxybenzaldehyde	1119, 2.420	1258.0	-	-	-	0.02±0.00	0.05±0.01
188	(Z)-2-Decenal	1128, 1.450	1262.8	-	-	0.02±0.00	0.01±0.00	-
189	Citral	1143, 1.630	1271.8	-	-	0.05±0.01	-	-
190	2,4-Decadienal	1182, 1.550	1295.0	-	-	0.24±0.04	0.13±0.01	0.10±0.06
	Subtotal			44.63	16.7	14.22	25.90	20.06
Ketones (8)								
191	Acetone	102, 0.600	500.3	-	0.29±0.02	1.35±1.02	-	1.12±0.99
192	Methyl vinyl ketone	120, 0.740	567.3	0.61±0.28	-	-	-	-
193	3-Octanone	636, 1.270	987.1	0.29±0.19	-	0.34±0.02	0.35±0.04	0.13±0.02

194	6-Methyl-5-heptene-2-one	639, 1.380	988.8	0.44±0.30	0.07±0.02	1.48±0.14	0.19±0.22	0.80±0.08
195	Acetophenone	786, 2.040	1068.4	0.13±0.01	-	0.20±0.02	-	-
196	(E)-4-Phenylbut-3-en-2-one	1200, 2.150	1306.5	-	-	0.03±0.00	-	-
197	(E)-6,10-dimethyl-5,9-undecadien-2-one	1431, 1.500	1456.3	-	-	0.59±0.03	0.11±0.02	0.36±0.13
198	4,4-Dimethyl-3-phenyl-2,5-cyclohexadien-1-one	1614, 1.830	1583.5	0.10±0.03	-	-	0.03±0.00	-
	Subtotal			1.57	0.36	3.99	0.68	2.41
Alcohols (20)								
199	Ethanol	90, 0.600	456.0	26.06±10.27	12.34±1.25	27.35±8.83	24.15±7.38	16.15±5.27
200	1-Propanol	108, 0.670	522.7	0.42±0.19	-	0.55±0.03	0.27±0.04	0.29±0.05
201	2-Methylpropanol	144, 0.750	624.1	-	-	-	0.18±0.03	-
202	1-Butanol	159, 0.820	648.0	2.66±0.25	1.50±0.54	8.80±1.02	10.20±0.94	5.55±0.50
203	2-Methylbutanol	234, 0.920	731.0	4.06±0.44	0.03±0.01	0.44±0.07	0.61±0.04	0.21±0.04
204	1-Pentanol	270, 0.990	766.9	-	0.11±0.03	0.43±0.12	0.38±0.04	0.19±0.03
205	1-Hexanol	420, 1.180	866.3	9.84±7.29	11.26±2.67	26.97±4.90	14.30±1.13	13.31±2.54
206	trans-2-Hexen-1-ol	420, 1.210	866.3	-	0.50±0.09	-	-	-
207	1-Heptanol	606, 1.200	970.7	0.16±0.02	0.02±0.00	0.25±0.04	0.17±0.01	0.13±0.03
208	2-Ethylhexanol	717, 1.190	1030.8	-	0.06±0.03	-	-	-
209	1-Octanol	792, 1.260	1071.2	0.57±0.18	-	0.99±0.38	0.44±0.04	0.43±0.13
210	2-Nonanol	846, 1.200	1100.2	-	0.08±0.02	0.19±0.05	-	-
211	Linalool	846, 1.260	1100.2	-	-	0.05±0.00	-	-
212	(E)-2-Nonen-1-ol	969, 1.340	1171.4	-	-	-	0.08±0.01	-
213	1-Nonanol	972, 1.290	1169.7	-	-	0.25±0.08	0.23±0.06	-
214	3,4,5-Trimethyl-4-heptanol	1044, 1.380	1212.7	0.90±0.21	-	-	-	-
215	Citronellol	1071, 1.370	1228.8	-	-	0.08±0.03	0.09±0.03	0.06±0.01
216	(Z)-4-Decen-1-ol	1119, 1.380	1257.4	-	-	0.03±0.01	-	-
217	1-Decanol	1143, 1.290	1271.6	-	-	0.05±0.00	-	-
218	2,3-Dihydrofarnesol	1764, 1.460	1693.6	-	-	-	0.09±0.02	0.07±0.02
	Subtotal			44.67	25.90	66.43	51.19	36.39
Others (23)								
219	Dimethyl sulfide	108, 0.600	522.4	-	0.19±0.04	-	0.53±0.51	0.23±0.08
220	2,3-Dihydrofuran	120, 0.640	567.2	0.04±0.01	0.03±0.01	-	0.10±0.01	0.16±0.04
221	Acetic acid	123, 0.690	578.2	-	-	-	0.06±0.01	0.07±0.00
222	2-Methylfuran	132, 0.690	609.7	0.48±0.50	-	0.65±0.22	-	0.11±0.04
223	Thioacetic acid	213, 0.680	718.0	1.40±0.13	-	-	-	-
224	Anisole	507, 1.550	918.4	0.02±0.00	-	0.05±0.01	0.03±0.00	0.03±0.01
225	Benzonitrile	633, 2.050	987.5	0.23±0.02	-	0.38±0.08	-	-
226	Phenol	639, 1.610	993.8	0.07±0.01	-	0.10±0.03	-	0.04±0.00
227	2-Pentylfuran	645, 1.160	993.6	0.26±0.04	0.33±0.07	4.86±0.32	4.45±0.88	2.61±0.39
228	1-Methoxy-4-methylbenzen	702, 1.580	1023.0	-	-	0.06±0.01	0.03±0.00	0.05±0.02

	e							
229	Eucalyptol	720, 1.230	1032.4	0.89±0.49	-	-	-	-
230	1-Methoxymethyl-2-methyl benzene	831, 1.640	1092.3	-	-	0.05±0.00	-	-
231	(Z)-Rose oxide	867, 1.290	1112.1	-	-	-	0.06±0.01	0.03±0.00
232	(E)-Rose oxide	897, 1.310	1129.0	-	-	-	0.05±0.00	-
233	β-Citronellol, methyl ether	984, 1.140	1178.1	-	-	-	0.11±0.01	0.06±0.02
234	Estragole	1023, 1.730	1200.5	2.03±0.45	-	0.41±0.20	0.02±0.00	0.07±0.02
235	(Z)-1-Methoxy-4-(prop-1-en -1-yl)benzene	1116, 1.810	1255.9	0.02±0.00	-	-	-	-
236	Anethole	1170, 1.870	1288.0	0.11±0.01	-	0.03±0.02	0.02±0.00	0.02±0.00
237	(E)-3-Octenoic acid	1320, 1.350	1382.9	-	-	-	0.11±0.01	0.08±0.02
238	Methyleugenol	1359, 1.990	1408.7	0.09±0.02	-	0.66±0.16	-	-
239	(Z)-1,2-Dimethoxy-4-(prop-1-en-1-yl)benzene	1497, 2.100	1500.8	-	-	0.05±0.02	-	-
240	Sesquirosefuran	1578, 1.440	1557.7	-	-	0.09±0.01	0.06±0.00	0.05±0.03
241	(E)-3-(4,8-Dimethylnona-3,7 -dien-1-yl)furan	1605, 1.420	1576.9	-	-	0.09±0.03	0.40±0.04	0.22±0.04
	Subtotal			5.64	0.55	7.48	6.03	3.83
	Total			838.12	208.45	511.87	379.72	328.02

[1] R.T. refers to the retention time. [2] RI refers to the calculated retention index. [3] Data are means±SD of three replications. [4] "-" represents no data.

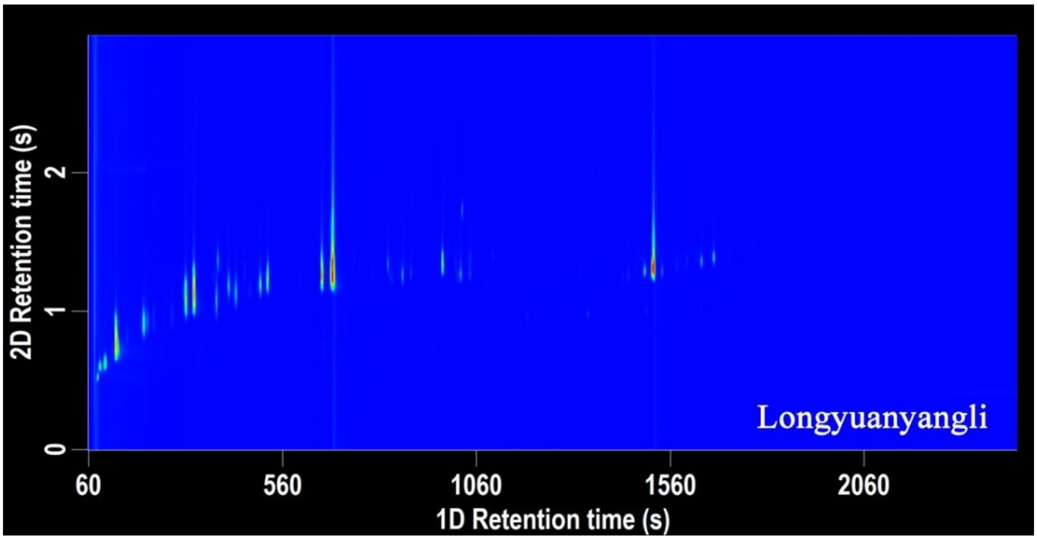

Figure S1. 2D chromatogram (total ion chromatography) of Longyuanyangli.

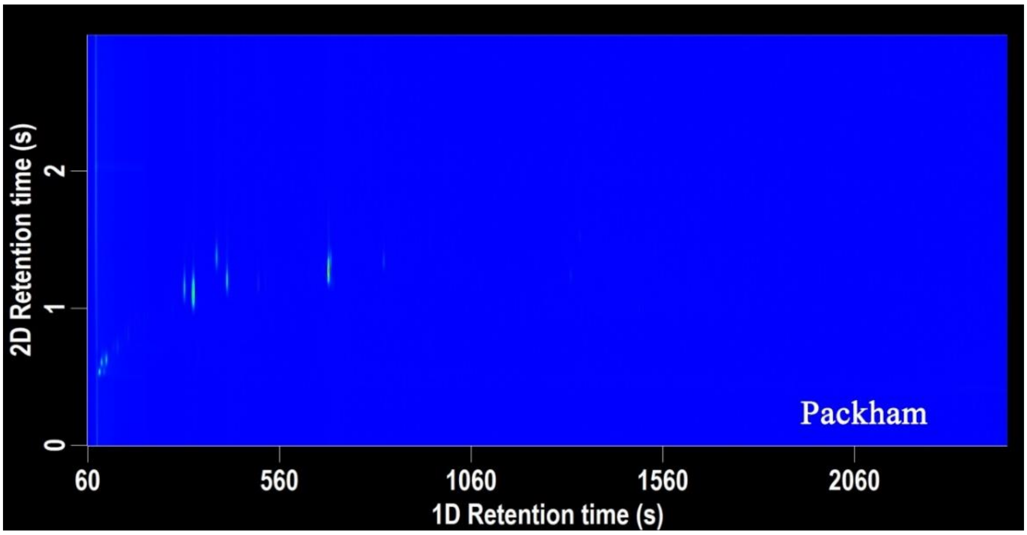

Figure S2. 2D chromatogram (total ion chromatography) of Packham.

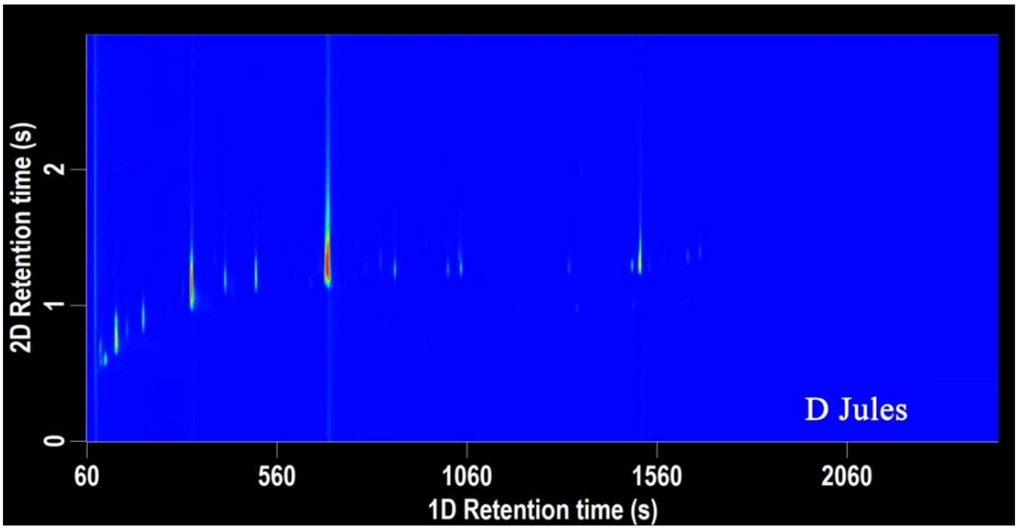

Figure S3. 2D chromatogram (total ion chromatography) of D Jules.

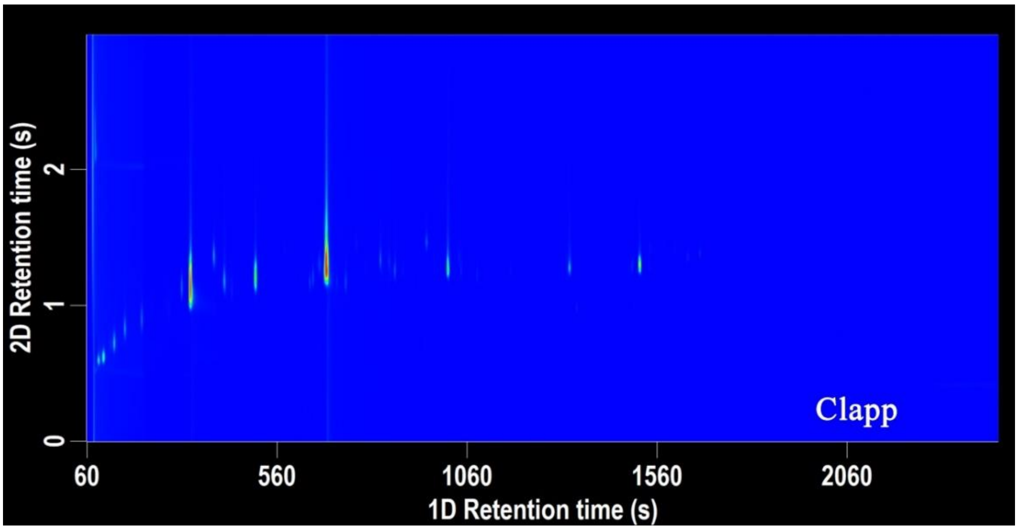

Figure S4. 2D chromatogram (total ion chromatography) of Clapp.

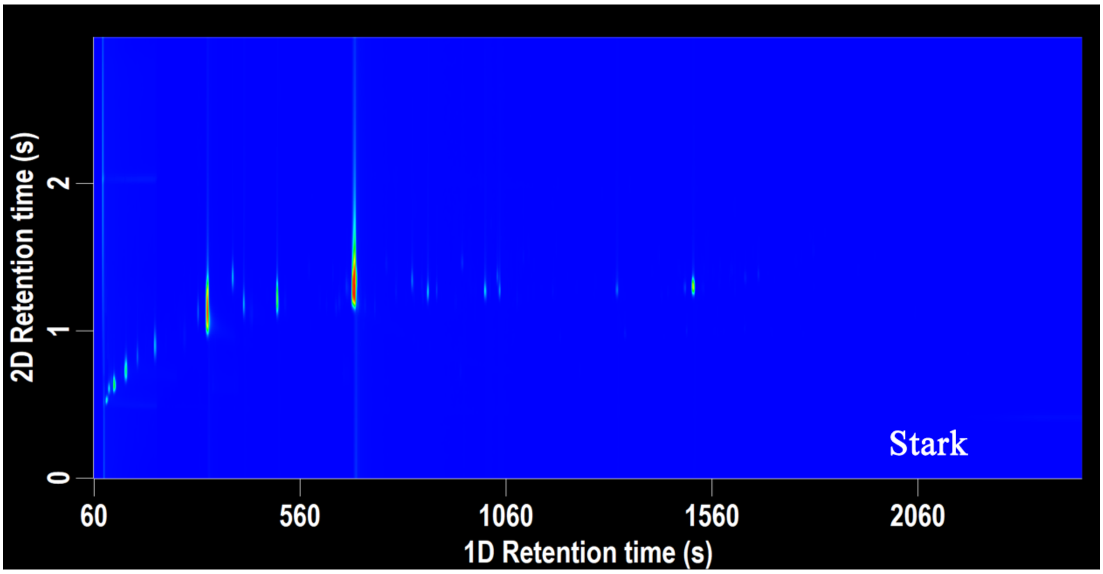

Figure S5. 2D chromatogram (total ion chromatography) of Stark.
